# EvoLaps 2: Advanced phylogeographic visualization

**DOI:** 10.1093/ve/vead078

**Published:** 2023-12-19

**Authors:** F Chevenet, D Fargette, P Bastide, T Vitré, S Guindon

**Affiliations:** MIVEGEC, IRD, CNRS, Université de Montpellier, Montpellier, France; PHIM, IRD, INRAE, CIRAD, Université de Montpellier, Montpellier, France; IMAG, CNRS, Université de Montpellier, Montpellier, France; MIVEGEC, IRD, CNRS, Université de Montpellier, Montpellier, France; LIRMM, CNRS, Université de Montpellier, Montpellier, France

**Keywords:** phylogeography, information visualization

## Abstract

EvoLaps is a user-friendly web application designed to visualize the spatial and temporal spread of pathogens. It takes an annotated tree as entry, such as a maximum clade credibility tree obtained through continuous phylogeographic inference. By following a ‘Top-Down’ reading of a tree recursively, transitions (latitude/longitude changes from a node to its children) are represented on a cartographic background using graphical paths. The complete set of paths forms the phylogeographic scenario. EvoLaps offers several features to analyze complex scenarios: (1) enhanced path display using multiple graphical variables with time-dependent gradients, (2) cross-highlighting and selection capabilities between the phylogeographic scenario and the phylogenetic tree, (3) production of specific spatio-temporal scales and synthetic views through dynamic and iterative clustering of localities into spatial clusters, (4) animation of the phylogeographic scenario using tree brushing, which can be done manually or automatically, gradually over time or at specific time intervals, and for the entire tree or a specific clade, and (5) an evolving library of additional tools. EvoLaps is freely available for use at evolaps.org.

## Introduction

Phylogeographic reconstructions are increasingly used to study the epidemiology and evolution of pathogens for effective public health measures and surveillance (Argimón et al. 2016; Bielejec et al. 2016; Dellicour et al. 2016; Hadfield et al. 2018; Nahata et al. 2022). Phylogeographic scenarios are generated by a root-to-tip reading of a phylogenetic tree annotated with discrete or continuous ancestral character states (locations) (Lemey et al. 2010), computed by Bayesian inference software programs like BEAST (Suchard et al. 2018) or BEAST2 (Bouckaert et al. 2019). Visualization of phylogeographic reconstructions may result in complex structures, where branches often become entangled, creating cluttered visuals that are difficult to interpret (Theys et al. 2019).

EvoLaps assembles numerical and graphical methods and tools into a user-friendly interface dedicated to the visualization and editing of evolutionary scenarios based on continuous phylogeographic reconstructions. EvoLaps is freely available for use at evolaps.org with online documentation at www.evolaps.org/evolapsdocs.

The first version of EvoLaps proposed functionalities for clustering locations and customizing phylogeographic visualization (Chevenet et al. 2021). EvoLaps 2 is the result of an extensive improvement and reshuffling of EvoLaps to cover the current needs of a large and growing community of users. It offers the following features to analyze complex scenarios:

- enhanced path display, using multiple graphical variables with time-dependent gradients;
- cross-highlighting and selection capabilities between the phylogeographic scenario and the phylogenetic tree;
- production of specific spatio-temporal scales and synthetic views through dynamic and iterative clustering of localities into spatial clusters;
- animation of the phylogeographic scenario using tree brushing, which can be done manually or automatically, gradually over time or at specific time intervals, and for the entire tree or a specific clade;
- an evolving library of additional tools, incorporating basic information visualization tools (e.g. migration distance over time, intensity map superimposed on the geographic map), along with more advanced tools, such as the maximum likelihood (ML) inference of ancestral character states on discrete variables, which enables the corroboration of the phylogeographic scenario with any phylogenetic trait history of interest, whether in ecology or epidemiology.

In the following, we illustrate the functionalities of EvoLaps through the analysis of two datasets related to the spatiotemporal spread of the rice yellow mottle virus (RYMV) in East Africa (Trovão et al. 2015; Hubert et al. 2017; Dellicour et al. 2018) and the West Nile virus (WNV) in North America (Pybus et al. 2012).

## EvoLaps interface

**Figure 1.**
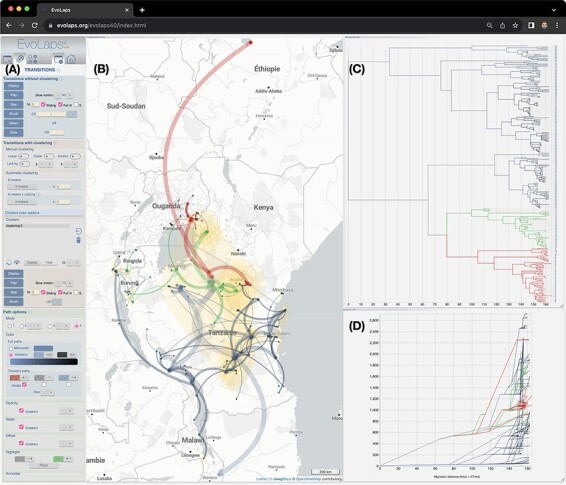
**Screenshot of the EvoLaps interface**. All interface controls are grouped on the left (**A**), within three toolboxes (‘Data’ for input/output management, ‘Transitions’ for the settings of the phylogeographic scenario, and ‘Edition’ for controls of all the other interface components such as the geographic map or the phylogenetic tree). The right part of the interface displays the components of the analysis: the phylogeographic scenario is depicted on a map with yellow areas indicating uncertainty zones associated with the most probable localities (**B**). Highlight colors (red, green) have been applied across the scenario, the phylogenetic tree (**C**), and an additional view displaying migration curves over time (preorder traversal of the phylogenetic tree, for each parent/child node pair: X-axis = cumulative branch length, Y-axis= cumulative great circle distances knowing latitudes and longitudes) (**D**). Each component frame is zoomable, movable, and resizable.

EvoLaps is a web-based application that has been rigorously tested and optimized for compatibility with various web browsers, including Chrome, Safari, Firefox, Edge, Opera, and Brave. On the one hand, the client-side of the application is primarily developed using HTML 5 and JavaScript, leveraging several libraries such as D3 and Leaflet for enhanced functionality. On the other hand, the server-side is built on a combination of PHP, Tcl, and Python scripts. As depicted in Fig. 1, the interface is organized into two primary sections: a left panel that houses the controls, and a right panel serving as the primary viewing area, which can be further divided into distinct zoomable, movable, and resizable components, as needed throughout the analysis. The controls are split into three distinct categories:

- ‘Data’ is a toolbox dedicated to managing the inputs and outputs of the interface. This includes features like importing annotated tree files in the NEXUS format, saving and restoring EvoLaps analyses (including demonstration data), and exporting graphics in Scalable Vector Graphics format.
- ‘Transitions’, a second toolbox, encompasses the display and editing tools for the evolutionary scenario. It also covers graphical parameters such as color settings and provides editing functionalities like selection and animation. Additionally, this toolbox offers tools for clustering both sampled and ancestral locations into spatial clusters.
- ‘Edition’, the third toolbox, gathers controls specific to each other components, such as the geographic map (e.g. tiles selection), the phylogenetic tree (e.g. tree layout, time scale), the transition tree, the transition charts, the migration curves over time, and the computation of ancestral character states from discrete variables.

## Display and edition of the phylogeographic scenario

Each node of the phylogenetic tree is represented on a cartographic background as a point located by its latitude and longitude coordinates (either ancestral/inferred or sampled/observed). By following a reading of the tree topology from the root to its leaves recursively, each transition, from a parent node to one of its children, is depicted using a graphical path object. The complete set of paths forms the phylogeographic scenario.

The display of paths is determined by four graphical variables: line thickness, curvature, opacity, and color. Each variable can be adjusted, allowing for the decrease or increase of line thickness, curvature, and opacity. Additionally, each graphical variable can have an optional time-dependent gradient that is based on the depth of the transition in the phylogenetic tree. For instance, if the line thickness gradient is activated, the maximum depth of the phylogenetic tree is defined (or proportional) to the paths from the root, and it decreases by one unit for its children at each recursive step. Adjusting the line thickness while the associated gradient is active adds or subtracts by one unit to all the transition paths in a relative manner. The phylogeographic scenario can be displayed using a single selected color or a gradient between two selected colors.

**Figure 2.**
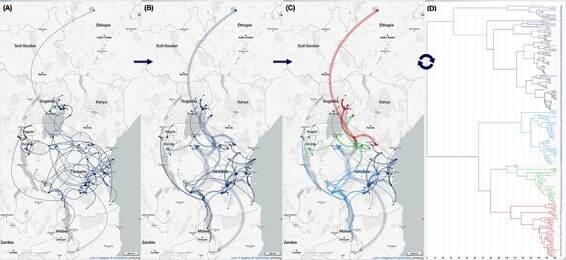
**Displaying a phylogeographic scenario of rice yellow mottle virus (RYMV) in East Africa**. Data from Trovão et al. (2015), Hubert et al. (2017), and Dellicour et al. (2018). **(A)** Original graphical representation. The phylogenetic tree is traversed recursively, starting from its root and moving towards its leaves. At each node transition, the associated coordinates are utilized to draw a migratory path on the geographical map. **(B)** The same phylogeographic scenario is displayed but with gradients linked to line thickness, curvature, opacity, and color. These gradients match the depth of nodes in the phylogenetic tree, ranging from ancient to recent: path thickness ranges from wide to narrow, curvature varies from high to low, opacity shifts from light to strong, and color transitions from light to deep yellow. Highlights and selections from the phylogeographic scenario (**C**) or the phylogenetic tree (**D**) are reflected in the phylogenetic tree or the phylogeographic scenario, respectively. Multiple selections with different colors (here red, green, and blue) help to decompose in time and space the evolving process to identify trends.

Fine-tuning these graphical variables can significantly enhance the readability of the phylogeographic scenario (Fig. 2A,B). For example, activating a curvature gradient in conjunction with a line thickness gradient makes the representation of the migration process akin to a bouncing ball, allowing for a better visualization of time-dependent spatial dynamics during the evolving process.

## Phylogeographic scenario highlights and selections

EvoLaps enables highlighting and cross-selection between the phylogeographic scenario and the phylogenetic tree. Pointing to a node in the tree highlights its origin all the way to the root, as well as the underlying clade, using two user-selected colors: an ‘upstream’ color and a ‘downstream’ color, respectively. This process automatically reflects on the phylogeographic scenario: the ‘upstream’ color overlays the phylogeographic scenario from its starting point to the selected node, while the associated migration paths of the clade are highlighted with the ‘downstream’ color. Conversely, pointing to a path in the phylogeographic scenario allows highlighting the upstream and downstream areas of the phylogeographic scenario, as well as the corresponding branches within the phylogenetic tree. Selection is performed using the same method, but with a mouse click, allowing the user to preserve the highlighted areas. The ‘upstream’ and ‘downstream’ colors can vary (user’s settings) between different selections. The pointing and selection capabilities facilitate the decomposition of the migration process and provide a clearer visualization of the interaction of the spatial and temporal dynamics (Fig. 2C,D). This option is particularly relevant in order to spot events whereby a dispersal event triggered speciation.

## Spatio-temporal scales and resolution of the phylogeographic scenario

**Figure 3.**
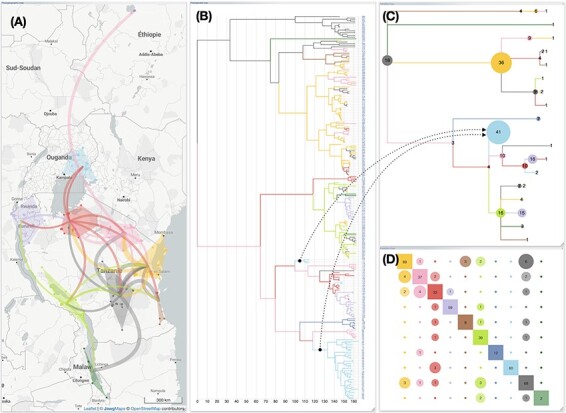
**Phylogeographic scenario with clustering of RYMV in East Africa**. Data from Trovão et al. (2015), Hubert et al. (2017), and Dellicour et al. (2018). The phylogeographic scenario solely based on a raw reading of ancestral and sampled localities is challenging to interpret (Fig. 2A). The grouping of ancestral and sampled localities into 10 regional clusters enables the inference of a simplified representation of the original scenario (**A**). The phylogenetic tree is colored according to this new spatial distribution **(B)**, and the corresponding inter-cluster transition tree (**C**) and transition chart (**D**) are computed. Here, the transition tree is a compressed layout showcasing nodes with size proportional to the numbers of taxa within the same cluster (for instance, the blue node has a surface proportional to the 3+ 38 = 41 taxa). The transition chart is a partial count from cluster X to cluster Y.

Rendering the phylogeographic scenario solely based on a raw reading of ancestral and sampled localities from the phylogenetic tree often leads to complex graphical representations (if n is the number of leaves of the tree, then the number of paths of the phylogeographic scenario is 2n–2). In order to provide users with a way to organize these complex structures in a meaningful way, EvoLaps incorporates a set of tools to group ancestral and sampled localities into spatial clusters of varying sizes (Fig. 3). Revisiting the phylogenetic tree and assigning continuous localities to spatial clusters yields an inter-cluster transition tree. Projecting these inter-cluster transitions onto the geographic map allows for a simplified version of the phylogenetic scenario. The degree of simplification depends on the number of clusters. Clustering is dynamic and iterative, so the users can define regions on the fly, save the output, and then further split a region into sub-regions to obtain more detailed information about a specific area in time or space, enabling a progressive exploration of phylogeographic scenarios. For instance, an analysis can be initialized with a small number of large clusters, and then selected clusters can be subdivided afterwards to achieve a higher resolution. The currently available clustering methods can be classified into two categories: highly interactive but manual methods, and automatic methods. Manual methods include lasso selection of localities on the geographic map, the use of draggable anchors, selection of clades from the phylogenetic tree, and the use of a latitude/longitude bounding grid with adjustable grid size and the ability to manually move each bounding point.

Clustering with anchors provides a straightforward and efficient method for assessing hypotheses of a phylogeographic scenario based on diverse spatial partitions. When this clustering mode is activated, clicking on the geographic map triggers the display of an anchor at the coordinates pointed to. Anchors serve as potential clusters and are movable (drag & drop) or removable (right-clicked). Their colors identify clusters, either chosen manually by the user or automatically assigned, based on the anchor coordinates at creation associated with a 2D color matrix selected by the user. The nearest localities to the anchor (measured by Euclidean distances) are colored with the anchor’s hue, indicating their cluster membership. Therefore, the user can move the desired number of dynamic anchors/clusters on the geographic map and, when satisfied, prompt the display of the corresponding phylogeographic scenario. Clusters are then represented as minimum convex hull polygons, with fill color specified by their original anchor.

Automatic clustering methods include the K-means method and the K-means method combined with bounding of latitude/longitude, using minimum and maximum values to define the bounds (which can be manually adjusted afterwards). The K-means algorithm (Hartigan et al. 1979) requires (a user’s setting) an initial number of seeds (cluster centroids) randomly generated within the boundaries of locations. Then, iteratively until no change, locations are assigned to their closest centroid based on the Euclidean distance, and centroids are updated. This algorithm can be used as a heuristic to automatically define geographically consistent groups, which can be adjusted by the user afterwards.

The inter-cluster transition tree is a multi-furcating tree-like representation capturing the essence of migrations. It offers a summarized view of the phylogeographic scenario, presenting a higher level of abstraction, depending on the number of clusters. This diagram can be further simplified by collapsing identical transitions that share the same ancestor in the original version of the transition tree (refer to Chevenet et al. 2021 for more details).

The interface also provides inter-cluster exchange maps, which are matrix representations that cross-reference clusters and count the number of transitions between each pair of clusters. These counts can be symmetric (from cluster X to Y and Y to X) or directed (from X to Y only). Users have the flexibility to order the rows and columns of the matrices in ascending order by latitude (rows) and longitude (columns). For instance, in Fig. 3B, clusters, distinguished by specific colors, are displayed using squares along the diagonal of the inter-cluster transition matrix. Numbers in the center of these squares are counts of intra-cluster transitions. Inter-cluster transitions are represented by circles of a color specific to the donor cluster. Numbers in the center of these circles are counts of transitions from this cluster to the cluster in the same row, along the diagonal. Thus, for a given cluster, the column of the associated matrix represents all emissions from that cluster, while its row indicates all migrations to this cluster. To enhance understanding, pointing to a circle in the transition matrix dynamically highlights on the geographical map, both the donor and recipient clusters and displays an arrow pointing from the source to the target clusters.

## Phylogeographic scenario animation

**Figure 4.**
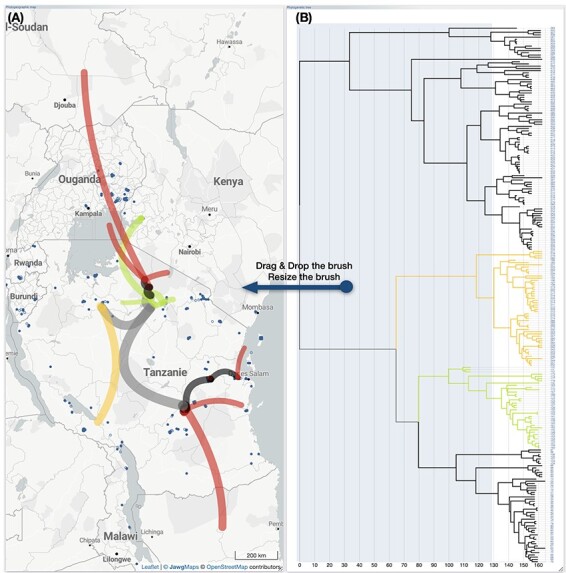
**Animation of the phylogeographic scenario by using a selection brush.** The animation of the evolutionary scenario, which aims to provide users with a dynamic view of the migratory process, involves the use of a selection brush on the phylogenetic tree and/or the inter-cluster transition tree. The selection brush, with adjustable position and size, allows for the selection of an origin point and a time window (represented, respectively, by the brush’s horizontal position and width); furthermore, the selection brush allows users to focus on a specific clade (its vertical position and height). The length of the transition paths is proportional to the branch length within the selection. The branches from parent to child nodes in the phylogenetic tree can be categorized into four groups based on whether the parent and child nodes fall within the selection window or not, and each category can be associated with a specific color. Here, transitions associated with the category ‘parent node selected/child node not selected’ are highlighted in red to facilitate the identification of migration starting points with the option to retain highlight colors.

The animation of the phylogeographic scenario, which involves generating a dynamic image, is a crucial element of the EvoLaps interface. This functionality allows for a better understanding of the spatio-temporal aspect of the migratory process. The animation of the phylogeographic scenario is achieved by using a selection brush on the phylogenetic tree and/or the transition tree (Fig. 4). The size and position of the selection brush are variable, either determined by the user on-the-fly or automatically by the interface. The use of a selection brush enables the display of a phylogeographic scenario limited to a specific clade or time slice, with the display of paths that may be partial. There are four possibilities: (1) the path between two points is ‘complete’ because the corresponding nodes within the phylogenetic tree are within the selection, (2) the path is ‘total partial’ because none of the associated nodes are within the selection, and the temporal selection window described by the selection brush lies between the two nodes (father vs son) of the tree, (3) the parent node is within the selection but not the child node, in which case the path is ‘downstream partial’, and (4) the child node is within the selection but not the parent node, and the path is ‘upstream partial’. The size of the partial paths is proportional to the branch length included in the selection, and complete downstream partial and upstream partial paths can be distinguished with different graphical characteristics, such as a specific color. This way, the user can sweep through the phylogenetic tree with a selection brush representing a specific time interval and visualize incoming migrations (upstream partial paths), outgoing migrations (downstream partial paths), or migrations in transit (total partial paths) more distinctly. Sweeping a brush over a narrow time selection window allows for the identification of rapid migrations by locating long partial paths. Furthermore, the selection can be focused on a specific clade, allowing for a subset of leaves to be targeted. The same approach has been adopted for spatial clusters, except that the selection brush is used on the transition tree.

The selection brushes can be controlled by the interface, based on user-defined parameters. For example, the width of the selection brush can be increased and it can sweep the tree at a given speed. The position of the selection brush can also be controlled by the interface to create a sliding window in time, effectively dividing the phylogeographic process into multiple consecutive stages (with an optional automatic graphical saving at each step).

## An evolving library of visual and analytical tools

EvoLaps is progressively incorporating new visualization and processing capabilities. For example, the ‘migration distance over time’ module significantly enhances the identification of slow and fast migrations (Fig. 1D). The phylogenetic tree is recursively read from the root to the leaves. For each parent/child node pair, a point is computed using the following coordinates: (X-axis = branch length between parent and child nodes, Y-axis = great-circle distance between parent and child nodes based on their spatial coordinates). This newly calculated point is then associated with the child node and visually connected in a cumulative way to the point associated with the parent node. As a result, the bundle of migration curves represents the phylogenetic tree, maintaining its topology, while assigning node ordinates based on their geographical coordinates. Importantly, highlights and selections from the migration distance curves are synchronized with the tree, the phylogeographic scenario, and vice versa.

**Figure 5.**
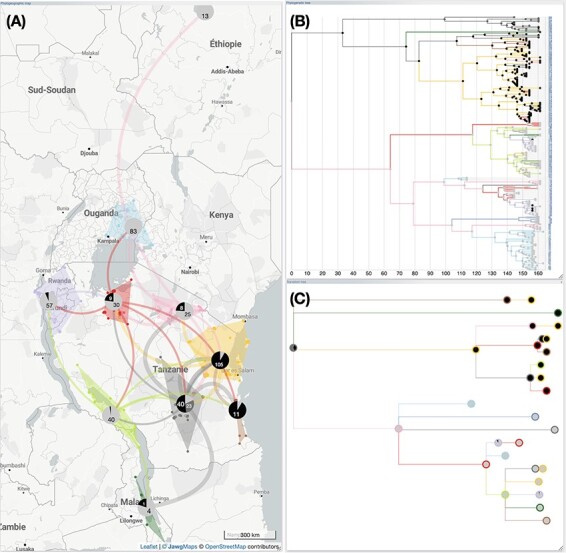
**Computing ancestral character states from a discrete variable.** In this example, Data from Trovão et al. (2015), Hubert et al. (2017), and Dellicour et al. (2018); the RYMV phylogeographic scenario in East Africa (clustered analysis) is juxtaposed with an amino acid insertion-deletion polymorphism at positions 18 and 61 of the RYMV capsid protein gene (for more details, see Abubakar et al. 2003). For this study, this polymorphism is categorized into three groups associated with the sequences: ‘S4’ (light grey), ‘S5’ (grey), and ‘S6’ (black). Ancestral categories are calculated using maximum likelihood with the F81 model, and they are visualized as pie charts superimposed on the phylogeographic scenario (**A**), the phylogenetic tree (**B**), and the transition tree (**C**). To achieve this, each node of the phylogenetic tree is analyzed, and a threshold for selecting the most probable ancestral categories is set; in this case, the threshold is the majority modality minus 20 per cent of its value. Then, the pie charts represent the count of the most probable ancestral categories retained for each group of nodes (cluster on the geographic map or node of the inter-clusters transition tree). This analysis highlights a decrease in polymorphism during the phylogeographic process, favoring the ‘S4’ form.

Another example is the computing and overlaying of ancestral character states from discrete variables. These variables can be of very diverse nature, such as insertion-deletion polymorphisms (as shown in Fig. 5), the emergence of treatment resistance in epidemiology, host–parasite interaction, or ecological traits (such as habitat type or index of environmental conditions favorable to a vector, etc.). Ancestral traits are computed using ML assuming an F81-like model of character evolution. A constant scaling factor is applied to all branch lengths to fit the rate of evolution of the studied character, with a value inferred by ML (Ishikawa et al. 2019). EvoLaps provides both the marginal posterior state probabilities (Felsenstein 1981) and the joint reconstruction with ML (Pupko et al. 2000). Results can be displayed on the phylogenetic tree, the transition tree, and the geographic map, considering a probability threshold beyond which the associated modality is retained. This threshold is the majority modality minus a percentage value set by the user.

## West Nile Virus in North America, a brief example of an EvoLaps analysis

The dataset comprises 104 WNV genomes collected between 1999 and 2008 from various US counties (Pybus et al. 2012). A step-by-step guide on reconstructing the spatial dynamics of WNV can be found at beast.community/workshop_continuous_diffusion_wnv. The output from TreeAnnotator (BEAST package) is then processed with EvoLaps.

**Figure 6.**
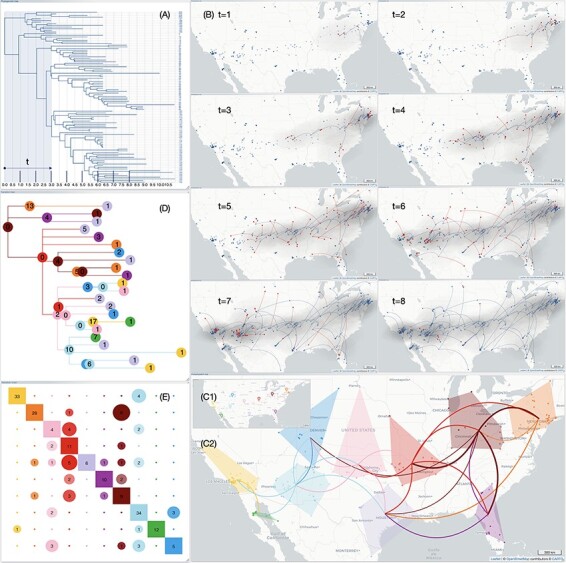
**Summary of an EvoLaps analysis related to the West Nile virus in North America**. Data from Pybus et al. (2012). Applying a sliding selection window to the phylogenetic tree (**A**) makes it easier to construct a panel representing different stages of the evolutionary scenario over time and space (**B,** t = 1 to t = 8). Ancestors or samples locations are depicted using empty or filled circles. Within these time intervals, emitting transitions are highlighted with a red head. The use of anchors on the geographic map (**C1**) allows for the dynamic and rapid definition of 10 regions (**C2**), simplifying the scenario from 206 transitions in the phylogenetic tree to 38 transitions in the transition tree (**D**). The transition chart (**E**) helps identify geographic areas in pink, red, maroon, and light blue as the most emitting, while the light purple area is the most receptive in terms of the diversity of source origins.

The application of a selection brush (Fig. 6A) across eight time frames provides a clear visualization of the successive stages of the dispersal in both time and space (Fig. 6B, t = 1 to t = 8). Early-stage short-distance migrations within the New York City area are succeeded by long-distance dispersal events towards the West and South, as depicted in EvoLaps with partial transitions highlighted with a red head.

A clustering with 10 anchors placed on the geographic map (Fig. 6C1) significantly simplifies the scenario (Fig. 6C2). The transition tree (Fig. 6D) represents a graphical compression of 81 per cent (from 206 transitions in the phylogenetic tree, to 38 transitions in the transition tree with the 10 areas). The associated transition chart (Fig. 6E) allows for the identification of the region extending from Phoenix to Santa Fe (light blue cluster), to be subjected of numerous short-distance migrations (34 intra-cluster transitions), and as the origin of numerous varied long-distance migrations: to the West (4 transitions to the yellow cluster), to the East (2, pink), to the North (3, dark blue), to the Southwest (1, green), and to the Southeast (2, light violet). Pink, red, and maroon regions also exhibit high emission levels. Intra-cluster transitions are also high for the yellow and orange regions. The light purple cluster appears to be the most diversified area in terms of incoming migrations (from six different clusters).

## Conclusion

EvoLaps provides researchers with a comprehensive and user-friendly interface for the analysis and visualization of phylogeographic scenarios. The software offers a range of features including customizable graphical parameters, time-dependent gradients, highlighting and cross-selection between the phylogeographic scenario and the phylogenetic tree, spatial clustering capabilities with production of synthetic views such as the transition trees or charts, and dynamic animation based on brushing processes. These features enhance the understanding of the spatio-temporal aspects of migratory processes. EvoLaps continues to evolve by incorporating new analytical modules to further enhance its display and editing functionalities. For instance, the data importation process will undergo a thorough revision in the upcoming versions to allow for the consideration of various data sources and formats. This will include a validation or specification step for correspondences between identified variables and the values expected by EvoLaps. A potential development could involve the ability to overlay multiple phylogeographic scenarios derived from several maximum clade credibility trees of the same continuous phylogeographic analysis. This would aid in identifying trends that are more or less supported by the data. Another potential future development could involve the integration of methods from the phylodynamic research field. For instance, enabling the visualization of velocities along spatial coordinates of present and past lineages. By combining these existing and potential features, EvoLaps empowers researchers to explore, analyze, and visualize phylogeographic scenarios in a detailed manner. This facilitates a deeper understanding of spatio-temporal evolutionary processes and interactions between populations.

## Data Availability

License: GNU GPL. Any restrictions to use by non-academics: none. EvoLaps source code and the datasets used in this study are available on http://www.evolaps.org/.
